# Human-Mouse Chimerism Validates Human Stem Cell Pluripotency

**DOI:** 10.1016/j.stem.2015.11.017

**Published:** 2016-01-07

**Authors:** Victoria L. Mascetti, Roger A. Pedersen

**Affiliations:** 1 The Anne McLaren Laboratory, Wellcome Trust-Medical Research Council Cambridge Stem Cell Institute, Department of Surgery and British Heart Foundation Centre of Regenerative Medicine, University of Cambridge, Cambridge, CB2 0SZ, UK

## Abstract

Pluripotent stem cells are defined by their capacity to differentiate into all three tissue layers that comprise the body. Chimera formation, generated by stem cell transplantation to the embryo, is a stringent assessment of stem cell pluripotency. However, the ability of human pluripotent stem cells (hPSCs) to form embryonic chimeras remains in question. Here we show using a stage-matching approach that human induced pluripotent stem cells (hiPSCs) and human embryonic stem cells (hESCs) have the capacity to participate in normal mouse development when transplanted into gastrula-stage embryos, providing in vivo functional validation of hPSC pluripotency. hiPSCs and hESCs form interspecies chimeras with high efficiency, colonize the embryo in a manner predicted from classical developmental fate mapping, and differentiate into each of the three primary tissue layers. This faithful recapitulation of tissue-specific fate post-transplantation underscores the functional potential of hPSCs and provides evidence that human-mouse interspecies developmental competency can occur.

## Highlights

• hiPSCs and hESCs form human-mouse interspecies chimeras with high efficiency
• hPSCs colonize gastrula-stage embryos in a manner predicted by fate mapping
• Integrated human cells disperse widely and express relevant differentiation markers
• Human-mouse chimeras provide in vivo functional validation of hPSC pluripotency

## Graphical Abstract

**Figure:**
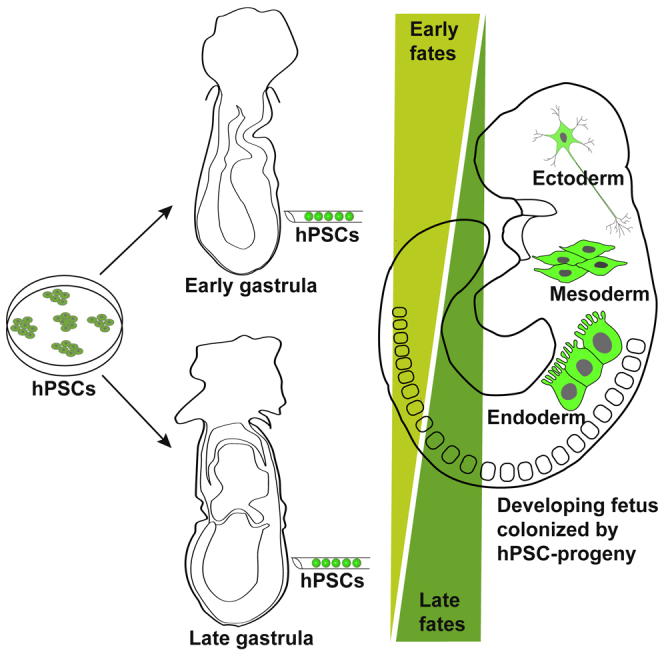

## Teaser

Mascetti and Pedersen show that stage-matching of human pluripotent cells to gastrula-stage mouse embryos overcomes barriers to chimerism, allowing efficient interspecies chimera formation with widespread cell dispersal and tissue-specific differentiation. These findings provide in vivo functional validation of hPSC pluripotency.

## Main Text

Human pluripotent stem cells (hPSCs) are characterized by biological properties similar to mouse epiblast stem cells (EpiSCs) but distinct from inner cell mass-like (ICM-like) “naïve” mouse embryonic stem cells (mESCs) (Mascetti and Pedersen, 2014). As such, hPSCs represent an epithelial epiblast-like state of pluripotency (Krtolica et al., 2007), commonly known as “primed.”

For mESCs, confirmation of stem cell pluripotency includes a demonstration of their ability to integrate into the preimplantation embryo and subsequently contribute to all the tissues of the developing mouse chimera (Bradley et al., 1984, Nagy et al., 1993). Interestingly, epithelial epiblast-like PSCs (such as mEpiSCs, hESCs, and hiPSCs), unlike their ICM-like counterparts (e.g., mESCs and miPSCs), are barely able to form preimplantation chimeras (James et al., 2006, Brons et al., 2007, Tesar et al., 2007, Masaki et al., 2015, Chen et al., 2015). However, mEpiSCs, which resemble the post-implantation epiblast, instead form chimeras with the post-implantation mouse embryo (Huang et al., 2012, Kojima et al., 2014). This raises a pivotal question: are hPSCs capable of forming an interspecies chimera by integrating into the post-implantation mouse embryo?

**Figure 1:**
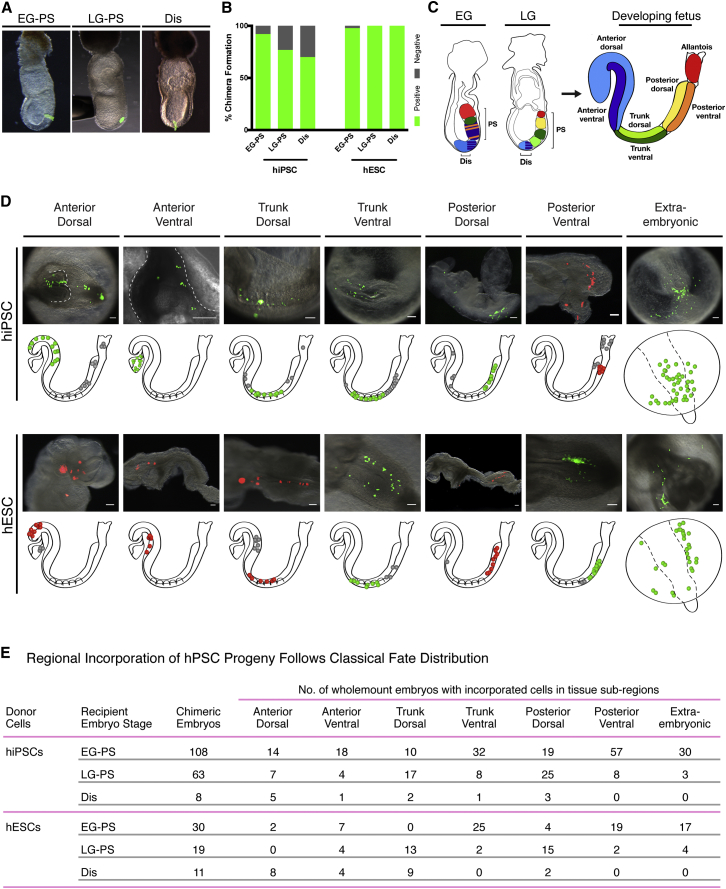
hPSCs Form Interspecies Chimeras with High Efficiency and Contribute to All Regions of the Developing Fetus (A) Representative image of hPSCs (hiPSCs or hESCs) constitutively expressing a fluorescent reporter transgene (green) transplanted to the Early gastrula primitive streak (EG-PS, left panel), Late gastrula primitive streak (LG-PS, center panel), or Distal tip (Dis, right panel) of gastrula-stage mouse embryos (shown before culture). (B) hiPSC- and hESC-transplanted embryos showed high incidences of chimera formation. EG-PS, Early gastrula primitive streak; LG-PS, Late gastrula primitive streak; Dis, Distal. (C) Incorporation of transplanted hPSCs during mouse gastrulation was assessed in relation to the predictive fate map of the embryo. The contribution of endogenous cells of early and late gastrula-stage embryos to the developing fetus predicts the fate of hPSC graft progeny. Schematics linking primitive streak (PS) and distal (Dis) sites of early gastrula (EG) and late gastrula (LG) stage embryos with their fate in the developing fetus (early somite stage) are shown. Subregions: red, extra-embryonic mesoderm (allantois and yolk sac); dark green, trunk ventral, including lateral plate mesoderm and mid-gut endoderm; purple, anterior ventral, including foregut endoderm, heart, and anterior neural crest; orange, posterior ventral, including hindgut endoderm; light blue, brain and surface ectoderm; and yellow, posterior dorsal, including presomitic mesoderm. See also Figure S1 for a representative image of fetus after culture. (D) Representative wholemount overlays (bright field plus fluorescence) with matched schematics illustrating subregional locations of hiPSC (top) and hESC (bottom) graft progeny after culture. hPSC graft progeny predominantly colonized embryos as dispersed populations of fluorescent cells. Green or red dots in the schematics represent clusters of cells (not individual cells) located in the specified subregion; gray dots represent fluorescent clusters located outside the specified subregion. hiPSC lines are shown as follows: FiPS, Posterior ventral; A1ATD-1, Anterior dorsal (aerial dorsal view, Anterior dorsal subregion outlined), Trunk dorsal, Trunk ventral, Posterior dorsal, and Extra-embryonic; and BBHX8, Anterior ventral (left side view of heart region outlined). hESC lines are shown as follows: H9, Trunk ventral, Posterior ventral, and Extra-embryonic; and Shef-6, Anterior dorsal, Anterior ventral, Trunk dorsal, and Posterior dorsal. Cluster score for each illustrated embryo was >4, except for Anterior ventral hESC embryo, which was 3. (E) Regional incorporation of hPSC progeny follows classical fate distribution. Summaries of number of chimeric embryos and subregional distribution of graft progeny following transplantation of hiPSCs (combined BBHX8, A1ATD-1, and FiPS data) and hESCs (combined H9 and Shef6 data) to the primitive streak (PS) of early gastrula (EG) and late gastrula (LG) stage mouse embryos or distal region (Dis) are shown. Embryos were scored as wholemounts for subregional incorporation, where graft progeny can colonize more than one subregion. See also Table S1 for data of individual hiPSC and hESC lines.

Based on these prior observations, we hypothesized that stage-matching hPSCs with their appropriate embryonic context would hold the key to unlocking chimeric competency. The epithelial epiblast-like phenotype of hPSCs, similar in nature to mEpiSCs, led us to predict that hPSCs would be able to form a chimera with the gastrula-stage mouse embryo. To test this idea, we transplanted three hiPSC and two hESC lines (together, hPSCs), each transfected with a fluorescent reporter gene, into early and late gastrula-stage mouse embryos at the primitive streak or distal tip of the epiblast (Figure 1A). We found highly efficient interspecies chimera formation in all transplant sites ranging from 70% to 100% of transplanted embryos following in vitro culture (Figure 1B). (Embryos were obtained and cultured under University ethical review according to UK animal regulations; see Supplemental Experimental Procedures.) We also transplanted mEpiSCs in a similar manner as a positive control for a putative interspecies barrier and saw similar incorporation (data not shown).

Classical fate mapping studies have established an experimental platform for assessing normal cellular participation during embryo development (Tam, 1989, Lawson et al., 1991). Accordingly, using these insights we developed a comprehensive allocation map that predicts the distribution of hPSC progeny from the transplantation site (primitive streak or distal) and stage (early gastrula or late gastrula) to seven subregional locations (Figure 1C). Together these subregions constitute the building blocks of the developing fetus, and contribution to them achieves embryonic, or primary, chimerism (McLaren, 1976).

We hypothesized that subregional cell fate could be used as a metric for normal participation of hPSCs during chimeric embryo development. We found that both hiPSC and hESC descendants had the capacity to colonize each of the subregions in the developing fetus during culture (Figure 1D). This ability of hPSCs to contribute to all subregions of the developing fetus is consistent with the classical definition of pluripotency. We also used specific prediction of graft allocation based on gastrula stage at transplantation to assay normal development of hPSC transplants in both early and late gastrulating embryos (Figure 1E). We found that subregional distribution of graft progeny was significantly different in early versus late gastrula primitive streak (PS) transplants for both hiPSCs and hESCs (hiPSC: X^2^, p = 0; hESC: X^2^, p = 0). Moreover, this significant difference was observed in each individual transplanted cell line, and we found no significant difference in graft progeny subregional distribution when comparing cell lines to each other (Table S1). More specifically, a comparison of gastrula transplant outcomes revealed that hPSCs transplanted to the early gastrula PS contributed significantly more to early patterned tissues (Anterior ventral, Trunk ventral, Posterior ventral, Extra-embryonic) when compared to later patterned tissues (Trunk dorsal, Trunk ventral, Posterior dorsal, Extra-embryonic) (hiPSC: X^2^, p = 0.006; hESC: X^2^, p = 0.003). The converse is also true: late tissue outcomes were predominantly derived from late gastrula PS transplants (hiPSC: X^2^, p = 7.4 × 10^−7^; hESC: X^2^, p = 0). These outcomes are propelled by the differences between total ventral and dorsal tissue allocation at gastrula stages: comparison of hPSCs transplanted to early versus late gastrula PS revealed a highly significant shift from ventral to dorsal fates as gastrulation progressed (hiPSC: X^2^, p = 1.6 × 10^−4^; hESC: X^2^, p = 0), as calculated by summing ventral and dorsal fates for all regions (anterior, trunk, posterior). More specifically, we observed a shift from Trunk ventral to Trunk dorsal (hiPSC: X^2^, p = 0.000362; hESC: X^2^, p = 1 × 10^−8^) and Posterior ventral to Posterior dorsal (hiPSC: X^2^, p = 7 × 10^−7^; hESC: X^2^, p = 9.19 × 10^−6^), but not Anterior ventral to Anterior dorsal, when comparing early to late gastrula PS transplants. Distal transplants did not significantly differ in relation to the gastrula stage of recipient embryos, as expected from predictive fate mapping, so we grouped distal transplants to the early and late gastrula for analysis. Subregional distribution of graft progeny from distal transplants differed significantly from PS transplants for both hiPSCs and hESCs (hiPSC: X^2^, p = 0.0107; hESC: X^2^, p = 4 × 10^−8^). More specifically, this is reflected in the divide between distal patterned tissues (Anterior dorsal, Anterior ventral) and PS patterned tissues (Anterior ventral-EG, Trunk dorsal, Trunk ventral, Posterior dorsal, Posterior ventral) (hiPSC: X^2^, p = 0.0132; hESC: X^2^, p = 2.66 × 10^−4^).

The hPSC patterning observed here reflects the progressive allocation of transplanted mEpiSCs (data not shown), resident epiblast descendants (Lawson et al., 1991), and orthotopic gastrula transplantations (Tam and Zhou, 1996, Kinder et al., 1999). Comparing hPSC allocation and differentiation capacity with the intricate process of gastrulation, as previously established by classical fate mapping, provides a rigorous challenge of human stem cell pluripotency. Hence predictive fate, established independently, can be used to evaluate whether transplanted cells behave normally. Based on that assessment, the hPSC fates we observed not only confirm the integration of hPSCs into the developing fetus but also indicate formation of a developmentally normal interspecies chimera.

**Figure 2:**
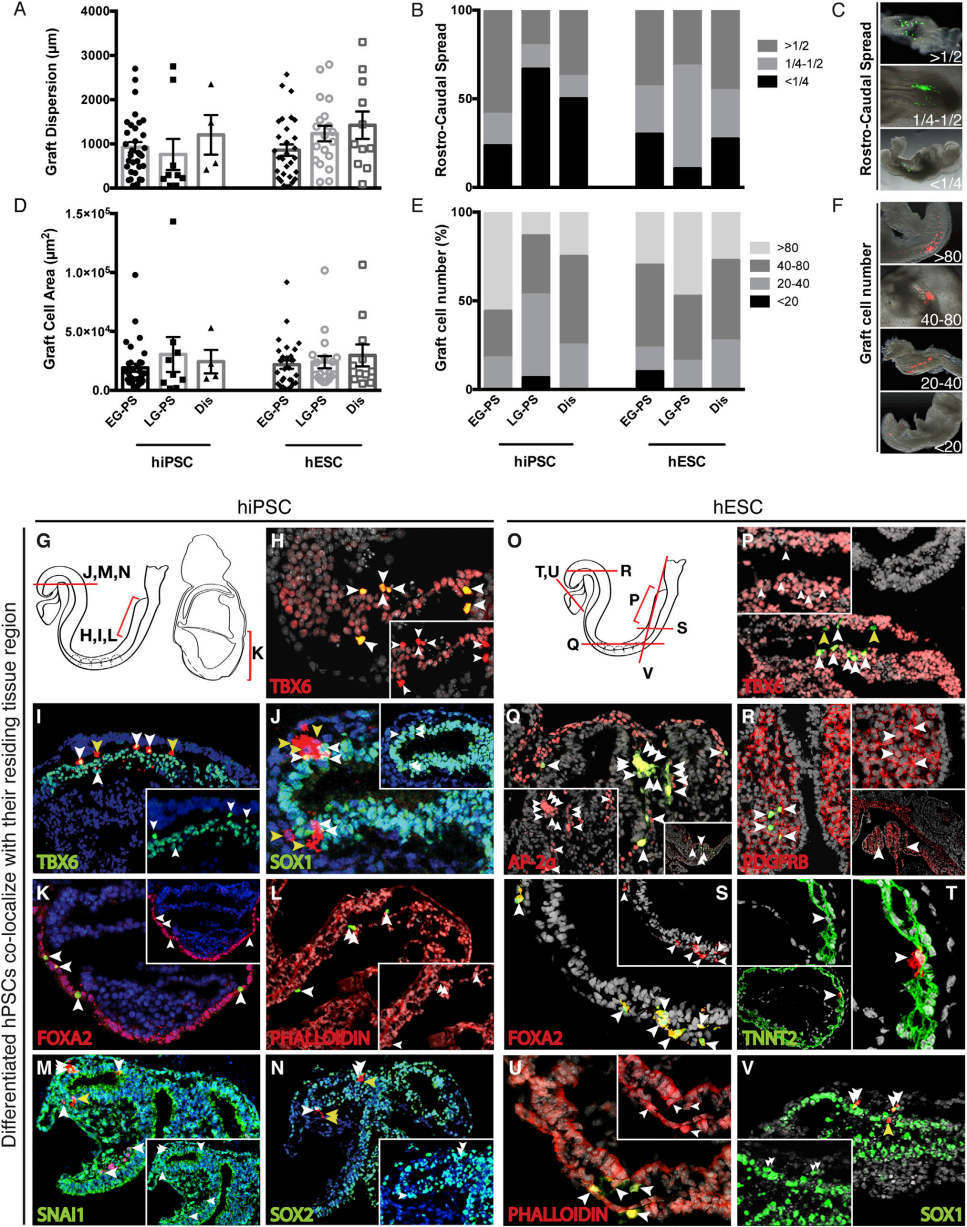
hPSC Graft Progeny Disperse in Host Embryos and Integrate within Their Residing Tissue Region (A–F) Dispersion of graft-derived cells in host embryos is indicative of proper tissue integration. Dispersion was assessed for both hiPSCs (A1ATD-1 and FiPS combined data) and hESCs (H9 and Shef-6 combined data) using the following parameters: graft linear dispersion; graft spread as a fraction of embryo rostro-caudal length; area occupied by graft cell descendants; and graft cell number. EG-PS, Early gastrula primitive streak; LG-PS, Late gastrula primitive streak; Dis, Distal. (A) hPSC progeny graft dispersion was measured along the greatest linear axis of the progeny population (see Supplemental Experimental Procedures). Graft progeny were extensively dispersed (mean ± SEM). (B) The extent of graft progeny spread was assessed as a fraction of embryo rostro-caudal length (<1/4, 1/4–1/2, or >1/2). (C) Examples of embryos with graft progeny spread of <1/4, 1/4–1/2, and >1/2. (D) Area covered by dispersed graft progeny (mean ± SEM). (E) Cell numbers were assessed in wholemount embryos as <20, 20–40, 40–80, or >80 cells. (F) Examples of embryos with <20, 20–40, 40–80, or >80 cells. (G–V) Differentiated hPSCs co-localize with their residing tissue region. Integration of hiPSC (G–N) and hESC (O–V) graft progeny was confirmed by immunostaining for specific tissue protein markers of the regions in which they resided. Co-localization of the fluorescent reporter and staining for marker gene expression in hPSC progeny (indicated by white arrowheads) confirmed tissue-specific gene expression; yellow arrowheads indicate non-co-localized graft progeny. Arrowheads are not representative of cell number. Insets show gene expression channel plus DAPI of region with hPSC progeny (with white arrowheads denoting hPSC progeny). (G) Schematic diagram of approximate section plane in hiPSC transplanted embryos at somite stage (left) or egg cylinder stage (right): (H), (I), (K), and (L), sagittal sections; (J), (M), and (N), cross sections. (H) TBX6 staining, showing nuclear localized H2B-Venus-expressing BBHX8 (BBHX8) progeny in presomitic mesoderm. (I) TBX6 staining, showing cellular localized Cherry-expressing FiPS (FiPS) progeny in presomitic mesoderm. (J) SOX1 staining, showing FiPS progeny in neuroectoderm. (K) FOXA2 staining, showing BBHX8 progeny in definitive endoderm of the egg cylinder. (L) Phalloidin staining, showing BBHX8 progeny in presomitic mesoderm. (M) SNAI1 staining, showing FiPS progeny in brain mesenchyme. (N) SOX2 staining, showing FiPS progeny in neuroectoderm. (O) Schematic diagram of approximate section plane in hESC transplanted embryos at somite stage: (P), sagittal sections; (Q)–(S), cross sections; (T)–(V), frontal sections. (P) TBX6 staining, showing cellular localized GFP-expressing H9 (H9) progeny in presomitic mesoderm. (Q) AP-2 alpha staining, showing H9 progeny in surface ectoderm. (R) PDGF receptor β staining, showing H9 progeny in branchial arch mesenchyme. (S) FOXA2 staining, showing H9 progeny in hindgut endoderm. (T) Troponin T staining, showing cellular localized tdTomato-expressing Shef-6 (Shef-6) progeny in heart. (U) Phalloidin staining, showing BBHX8 progeny in endoderm and mesoderm. (V) SOX1 staining, showing Shef-6 progeny in neuroectoderm. For (A)–(F), see also Table S2 for graft progeny rostro-caudal spread and cell number data of individual hiPSC and hESC lines. For (G)–(V), see also Figure S2 for separate channel images of each germ layer.

Strikingly, hPSC graft progeny, whether from early or late gastrula PS or distal sites, were frequently dispersed throughout and across embryonic subregions. This distribution reflects the normal dispersion of epiblast descendants during gastrulation and thus is evidence not only of cell integration but also of normal development (Lawson et al., 1991). Graft progeny dispersion, quantified as one-dimensional linear distance, averaged 963 μm and 1,171 μm for hiPSC and hESC transplants, respectively (Figure 2A), indicating extensive chimeric contribution. Graft progeny dispersion was also extensive in relation to embryonic rostro-caudal length whereby graft progeny colonized over one-fourth of the host embryo length in 70% of hiPSC chimeras and 77% of hESC chimeras (Figures 2B and 2C). We also quantified the cell area covered by dispersed graft progeny (Figure 2D). Total estimated graft cell number (Figures 2E and 2F) indicated that transplanted donor cells (∼10) proliferated extensively (hiPSC: mean = 70; hESC: mean = 64). Estimated cell numbers overlapped the ranges found for orthotopic transplants of comparable numbers of embryonic epiblast cells (proximal transplants, 70–77 cells /embryo; Tam and Zhou, 1996) and for orthotopic transplants of PS cells (33–78 cells/embryo; Kinder et al., 1999). For the purposes of dispersion analysis, we quantified the number of separate cell clusters, as distinct from a single cluster (which could result from formation of an intra-embryonic teratoma by aberrant cell growth). Multiple (≥2), dispersed clusters were the predominant outcome in both early and late gastrula transplants for both hiPSCs (96%) and hESCs (91%) and for individual cell lines (Figures S2A and S2B). We also found no significant difference in graft progeny dispersion metrics when comparing hPSC cell type (hiPSC versus hESC) or transplant sites (Early gastrula versus Late gastrula versus Distal). Thus, we conclude that transplanted hPSCs and their progeny proliferate and contribute normally to the developing embryo, irrespective of transplant stage, site, or cell type.

Confirming the localization and dispersion analysis, hiPSC and hESC graft progeny differentiated into endoderm, mesoderm, or ectoderm as shown by marker gene expression. We found that transplanted hiPSCs and hESCs co-expressed markers of the tissue in which they resided (hiPSC: Figures 2G–2N; hESC: Figures 2O–2V), indicating that they have undergone location-appropriate rather than random differentiation. Graft progeny located outside the antibody-designated tissue regions did not express the marker proteins, again showing that they were not randomly differentiated. Marker staining therefore also supports our conclusions that transplanted hiPSCs and hESCs differentiate to the phenotype corresponding to their local environment.

In summary, we have shown that stage-matching PSC types with their appropriate embryonic context can overcome barriers to chimeric competency. Our approach was based on a prediction that epithelial epiblast-like hPSCs would integrate efficiently into gastrula-stage embryos. This stage-matching concept explains the diversity of chimeric outcomes seen in previous studies. ICM-like mESCs, mouse iPSCs, rat iPSCs, and naive-like monkey ESCs form preimplantation chimeras (Bradley et al., 1984, Okita et al., 2007, Wernig et al., 2007, Buehr et al., 2008, Chen et al., 2015). However, epithelial epiblast-like hPSCs do not share this preimplantation chimeric competency, as shown in a recent report assessing hPSCs as non-chimera-forming cells (Masaki et al., 2015), likely because they represent a post-implantation cellular phenotype (Mascetti and Pedersen, 2014). Consistent with this, we found that when stage-matched to the epiblast of the gastrulating embryo, mEpiSCs (data not shown; Huang et al., 2012, Kojima et al., 2014), hiPSCs, and hESCs (as shown herein) demonstrate efficient chimeric formation not limited by transplant site. Most importantly, our findings in conjunction with previous studies indicate that chimeric competency relies on pairing in vitro derived cells with their in vivo counterpart.

Moreover, while alternative human pluripotent states may be demonstrable in vitro, if they are not as chimera competent as shown herein, our findings imply that they are either not in fact pluripotent or they have not been matched to the correct stage of embryonic development. We would therefore argue that their relevance remains inconclusive without confirmation of their contribution to organized in vivo differentiation in the context of normal embryogenesis. In this regard, our findings are distinct from a recent paper by Wu et al., which states that H9-hESCs do not integrate into the post-implantation mouse embryo (Wu et al., 2015). By contrast, we found highly efficient engraftment and extensive dispersion of graft progeny in each of the five hPSC lines transplanted, regardless of transplantation site (PS or distal tip), with integration and location-appropriate differentiation. The hESC mean linear dispersion that we observed was 40-fold and 84-fold greater for distal transplants, and 39-fold and 5-fold greater for LG-PS transplants than described by Wu et al. for H9-hESC and region selective-hESC dispersion, respectively. The technical challenges of the experimental system may be contributing factors in the disparity of the results. It is also possible that a “region-selective” cell type with restricted capacity is not in fact fully pluripotent, but instead is a partially differentiated cell that has been transplanted to its own permissive stage and location-matched environment.

Our demonstration that hPSCs contribute to multiple tissue layers reveals their in vivo pluripotency. This key observation has been lacking from previous in vitro assays, teratoma studies, and biased in vivo contribution, all of which are limited by their lack of organized tissue context and thus likely harbor artifacts. Evidence for hPSC pluripotency from in vitro differentiation and teratoma assays has been used to ascribe pluripotent status to both hPSCs and EpiSCs. Our approach goes beyond those assays by providing evidence for the capacity of hPSCs to participate in normal organized tissue development in an embryonic context. Contribution to terminally differentiated tissues would not be expected in the assay within the timeframe of embryo culture. Instead, the strength of our functional evidence for hPSC pluripotency is underpinned by normal mouse embryo development during this period (Figure S1). Our in vivo validation of hPSC developmental competency therefore provides a more rigorous demonstration of hPSC pluripotency than has been possible from prior approaches.

Moreover, our approach also confirms the capacity of hPSCs to participate in the intricately choreographed events of gastrulation by replicating the embryo’s own pluripotent cell migration and acquisition of specialized phenotypes. This also reveals that human pluripotent cells are able to correctly respond to the complex and progressively changing “permissive/stage-matched” signaling environment in which they reside after chimeric transplantation. As such, chimera competency with post-implantation mouse embryos underscores hPSC relevance for modeling human gastrulation in vitro, a developmental stage that would otherwise be inaccessible in vivo. Reciprocally, by mimicking the cues of gastrulation, we are able to extrapolate the conditions that pattern PSCs to specific tissue types in vitro (Mendjan et al., 2014).

In sum, faithful recapitulation of tissue-specific chimeric fate provides in vivo functional validation for human stem cell pluripotency. Our work provides evidence that hPSCs are not an artifact of culture and further endorses their utility as a bona fide resource for regenerative medicine.

## Author Contributions

V.L.M. obtained funding, designed and performed experiments, analyzed results, and wrote the paper; R.A.P. obtained funding, analyzed results, and wrote the paper.

## Supplemental Information

Document S1. Tables S1 and S2, Figures S1 and S2, and Supplemental Experimental Procedures

Document S2. Article plus Supplemental Information
