# Supplementary material for: Human-Mouse Chimerism Validates Human Stem Cell Pluripotency
Source: Cell Stem Cell. 2016 Jan 7;18(1):67–72. doi: 10.1016/j.stem.2015.11.017 (PMC4712187; doi:10.1016/j.stem.2015.11.017)
Supplement: Document S1. Tables S1 and S2, Figures S1 and S2, and Supplemental Experimental Procedures [file mmc1.pdf]

Cell Stem Cell
Supplemental Information

Human-Mouse Chimerism Validates Human Stem Cell Pluripotency

Victoria L. Mascetti and Roger A. Pedersen

Table S1. Regional Incorporation of hPSC Progeny by Line Follows Classical Fate Distribution

			No. of wholemount embryos with incorporated cells in tissue sub-regions						
Donor Cells	Recipient Embryo Stage	% Chimera Formation	Anterior Dorsal	Anterior Ventral	Trunk Dorsal	Trunk Ventral	Posterior Dorsal	Posterior Ventral	Extra-embryonic
BBHX8	EG-PS	76	6	8	5	14	6	30	12
	LG-PS	64	2	2	12	6	17	7	3
	Dis	40	3	0	0	0	1	0	0
A1ATD-1	EG-PS	100	4	10	3	13	12	21	11
	LG-PS	83	3	2	4	0	6	1	0
FiPS	EG-PS	100	4	0	2	5	1	6	7
	LG-PS	83	2	0	1	2	2	0	0
	Dis	100	2	1	2	1	2	0	0
H9	EG-PS	95	2	6	0	19	3	11	14
	LG-PS	100	0	1	7	1	10	1	3
	Dis	100	3	1	4	0	1	0	0
Shef6	EG-PS	100	0	1	0	6	1	8	3
	LG-PS	100	0	3	6	1	5	1	1
	Dis	100	5	3	5	0	1	0	0

Figure S1. Normally developing fetus after 2 day culture

A B

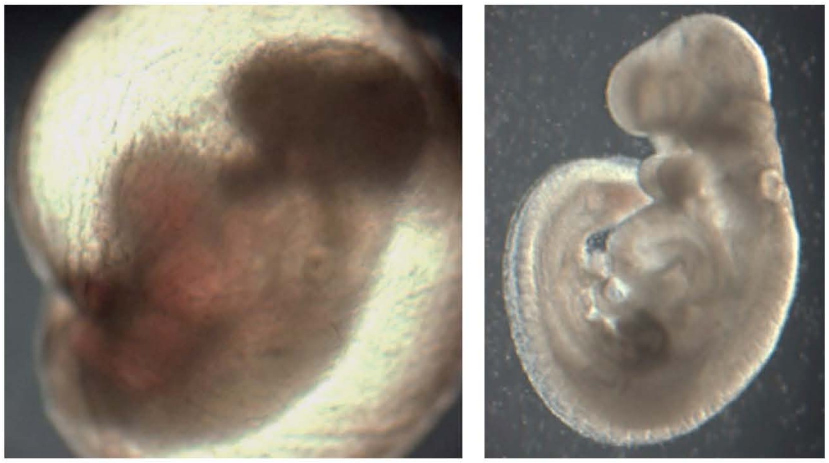

Table S1. Regional incorporation of hPSC progeny by line follows classical fate distribution (Related to Figure 1E).

Embryos were scored as whole mounts for sub-regional (Anterior Dorsal, Anterior Ventral, Trunk Dorsal, Trunk Ventral, Posterior Dorsal, Posterior Ventral) and extra-embryonic (allantois and yolk sac mesoderm) incorporation of hPSC progeny for each of the transplanted lines (hiPSCs: BBHX8, A1ATD-1, FiPS; hESCs: H9, Shef6). Results are shown for injections into early gastrula primitive streak (EG-PS), late gastrula primitive streak (LG-PS) and Distal tip (Dis).

Figure S1. Normally developing fetus after 2 day culture (Related to Figure 1)

Embryos cultured for 2 days following hPSC transplant developed normally.

Table S2. Extent of Graft Progeny Spread and Cell Number Distribution by hPSC Line

Donor Cells	Recipient Embryo Stage	Chimeric Embryos	Extent of Graft Spread			Graft Cell Number			
			<1/4	1/4-1/2	>1/2	<20	20-40	40-80	>80
BBHX8	EG-PS	74	37	27	10	51	19	4	0
	LG-PS	48	33	6	9	30	14	4	0
	Dis	4	4	0	0	4	0	0	0
A1ATD-1	EG-PS	24	4	5	15	0	3	4	17
	LG-PS	10	6	2	2	0	6	2	2
FiPS	EG-PS	10	4	1	5	0	3	5	2
	LG-PS	5	4	0	1	1	1	3	0
	Dis	4	0	1	3	0	1	2	1
H9	EG-PS	21	3	7	11	2	3	9	7
	LG-PS	12	2	8	2	0	2	3	7
	Dis	5	3	1	1	0	3	2	0
Shef6	EG-PS	9	6	1	2	1	1	5	2
	LG-PS	7	0	3	4	0	1	4	2
	Dis	6	0	2	4	0	0	3	3

Figure S2. hPSC Graft Progeny Disperse in Host Embryos and Integrate within Each Germ Layer

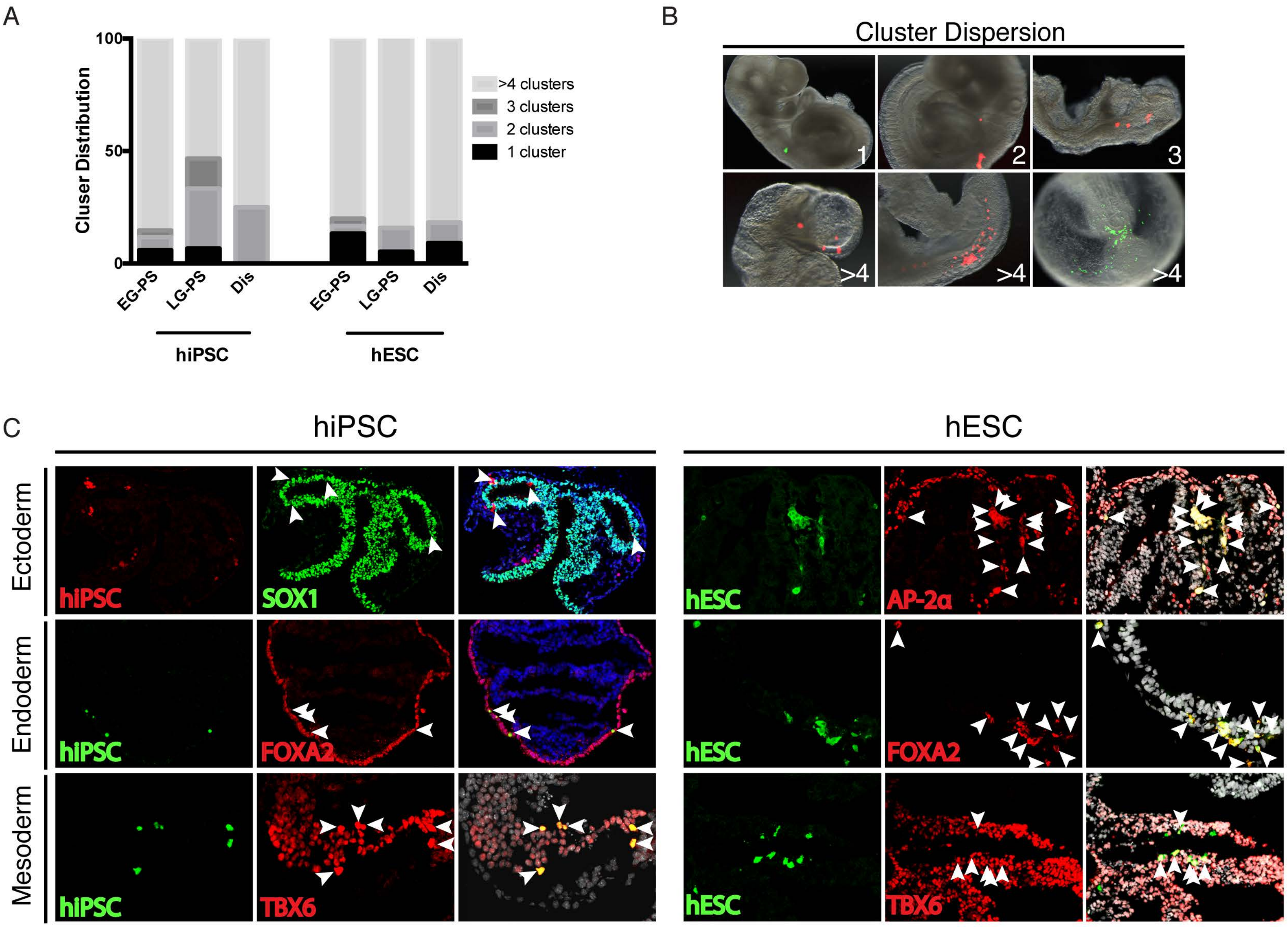

Table S2. Extent of graft progeny spread and cell number distribution by hPSC line (Related to Figure 2)

Embryos were scored as whole mounts for the extent of graft progeny spread (<1/4, 1/4-1/2 or >1/2, relative to the embryo's length) and cell number ranges (<20, 20-40, 40-80 or >80) for each of the transplanted hPSC lines (hiPSCs: BBHX8, A1ATD-1 and FiPS; hESCs: H9 and Shef6). Results are shown for injections into early gastrula primitive streak (EG-PS), late gastrula primitive streak (LG-PS) and Distal tip (Dis).

Figure S2. hPSC graft progeny disperse in host embryos and integrate within each germ layer. (Related to Figure 2)

(A) Embryos were scored as whole mounts for the number of spatially distinct clusters (1, 2, 3 or ≥ 4) summarizing hiPSCs and hESCs separately. Results are shown for injections into early gastrula primitive streak (EG-PS), late gastrula primitive streak (LG-PS) and Distal tip (Dis).

(B) Examples of embryos with graft cluster dispersion scores of 1, 2, 3 and >4.

(C) hiPSC graft progeny show co-localized gene expression within each germ layer. Illustrations of immunohistochemistry on cryosections with separated fluorescent channels for graft progeny and protein markers of germ layer in which they reside: ectoderm, endoderm or mesoderm. hiPSC staining for SOX1, marker of neuroectoderm; FOXA2, marker of endoderm; and TBX6, marker of presomitic mesoderm; hESCs, staining for AP-2alpha, marker of surface ectoderm; FOXA2, marker of endoderm; and TBX6, marker of presomitic mesoderm. In each case, the left panel shows the fluorescent hPSC progeny, the center panel the resident tissue

layer marker, and the right panel the merged channel. White arrowheads mark hPSC progeny (not representative of cell number).

Supplemental Experimental Procedures

Cell lines and culture

Human pluripotent stem cell (hPSC) lines BBHX8 (Cho et al., 2012), A1ATD-1 (Rashid et al., 2010) and H9 were provided by Dr L Vallier, University of Cambridge and cultured as previously described (Hannan et al., 2013). Briefly, these were grown in chemically defined medium (CDM) supplemented with Activin A (10ng/ml), Fibroblast Growth Factor (12ng/ml) and Insulin (7µg/ml) and 0.5% bovine serum albumin (BBHX8 and H9) or 1% polyvinyl alcohol (A1ATD-1). Passaging was done either using phosphate buffered saline (BBHX8) or collagenase/Dispase (H9, A1ATD-1) treatment. The BBHX8 hPSC line was transduced with a PiggyBac vector (gift of Dr Barry Rosen) encoding a nuclear localised Histone 2B-Venus fusion protein (H2B-Venus), and A1ATD-1 and H9 lines were transfected with a vector encoding cellular localized Green Fluorescent Protein (GFP). The Shef6 hESC line was provided by Dr Peter Andrews, University of Sheffield (Aflatoonian et al., 2010) and was transfected with cellular localized td-Tomato. The FiPS hiPSC line was provided by Dr Austin Smith, University of Cambridge (Takashima et al., 2014) and was transduced with cellular localized PB-Cherry. Both Shef6 and FiPS were grown in DMEM supplemented with Knockout Serum Replacer (KSR) and Fibroblast Growth Factor (4ng/ml for Shef6 and 10ng/ml for FiPS) and passaged using collagenase/Dispase.

Mouse embryo culture and injection

Embryos of CD1 mice mated *inter se* were obtained at either E6.5-6.75 or 7.5-7.75 staged according to Downs and Davies (Downs and Davies, 1993). Conceptuses were dissected in DMEM/F12 medium with Hepes (Gibco 31330-038) supplemented with

5% fetal calf serum, resulting in non-intact conceptuses. Early and mid-streak stages were grouped for injection as early gastrula, while late streak, no/early bud, and head fold stages were grouped as late gastrula. Injections were performed using Leica mechanical micromaniplators, Prime Tech Pmm4G piezoelectric injector, and Eppendorf Cell Tram syringes and bevelled microinjection needles (Eppendorf TransferTipES or Origio MSC-18-25). Colonies were partially dissociated by brief treatment with Ca^{++}- and Mg^{++}-free DPBS or PBS-based Cell Dissociation Buffer (Life Technologies), followed by trituration to obtain clumps of approximately 10 cells for injection. This number of transplanted cells was chosen to parallel mouse pre-implantation chimera approaches, and the relatively small cell number enabled assessment of developmental outcome at higher resolution. Graft cells were transplanted to the primitive streak (posterior and middle, grouped for analysis) and distal tip of the early gastrulating embryo and to the primitive streak (middle) and distal tip of the late gastrulating embryo. Distal transplants, having the same fate, were grouped for analysis. Embryos were cultured in DMEM/F12 + Glutamax and pyruvate (Gibco 10565-018) supplemented with 50% heat-inactivated rat serum (Harlan BT-4520). Early gastrula stage embryos were cultured at 37C in 5% CO_2 in air in static wells (Falcon 3037) for the first day, then transferred to fresh medium in rotating glass drums at 37C in 5% CO_2 + 20% O_2 (BTC Engineering Precision Incubator, Cambridge, UK) for the second day of culture. Late gastrula stage embryos were cultured in rotating drums for both days.

Chimeric embryos were assessed for normal development using previously established metrics as reference criteria (Beddington, 1981); (Theiler, 1989): General morphology, heart development and beating, yolk sac circulation, somite number, and

embryo turning. Only normally developed embryos were included in the analysis irrespective of chimera formation.

Embryos were scored for fluorescent progeny of injected cells using either a Leica fluorescence dissecting microscope (M165FC) or an Olympus inverted scope (IX71), fixed in 4% paraformaldehyde (2h to overnight, depending on stage) then washed and stored in PBS until embedded for histology.

The following scoring criteria were used to assess extent of colonization of the recipient embryos by fluorescent progeny of the injected cells: regional contribution was scored as anterior, trunk, posterior; and subregional contribution as dorsal or ventral in each case; or as extra-embryonic (yolk sac or allantois). Distribution of hPSC progeny to ventral and dorsal fates was calculated for whole subregional distribution by summing ventral versus dorsal fates for all regions (anterior, trunk and posterior). The extent of contribution was scored 1-4, where 1 = a cluster of contiguous cells; 2 = two clusters of cells separated from each other by > 1 cell diameter; 3 = three separated clusters of cells; >4 = four or more separated clusters of cells or cells in two different tissue regions or sub-regions. In addition, the approximate numbers of fluorescent reporter-labelled cells (<20, 20-40, 40-80 or >80) and extent of transplant progeny rostro-caudal spread (<1/4, 1/4-1/2, or >1/2 relative to embryo rostro-caudal length) were estimated live or in photographic images of whole mount embryos; for sectioned embryos, cell numbers were determined by cumulative cell counts. Measurements of linear dimension of graft progeny dispersion and area occupied by graft progeny were performed using the Leica Application Suite Interactive Measurement Module.

Histology and staining

Fixed embryos were embedded in OCT (VWR), frozen and cryosectioned at 6 μm, then stained for tissue markers as previously described (Fischer et al., 2008). For fixed frozen sections and wholemount embryos we used antibodies, which were selected as representative markers of the tissue layers or regions, to the following epitopes: Endoderm: FOXA2 (AF2400, R&D Systems); Neuroectoderm: SOX2 (SC17320, Santa C); SOX1 (AF3369, R&D Systems); Surface ectoderm: AP2alpha (5E4, Developmental Studies Hybridoma Bank); Mesoderm: Cardiac muscle: TNNT2 (MS-295-PO, Thermo Scientific); Presomitic mesoderm: TBX6 (AF4744, R&D Systems); Brain mesenchyme: SNAIL (ab180714, Abcam); Mesenchyme: PDGFRβ (ab88649, Abcam). In addition, Phalloidin staining was used to detect F-Actin (Sigma); anti-GFP (ab13970, Abcam) was used for Venus enhancement. Each antibody is reported by the manufacturer and confirmed in vitro to recognize both mouse and human epitopes. Secondary antibodies were donkey anti-goat, -mouse, -rabbit, and -chicken at 488, 568, 594 and 647 μm (Life Technologies). Black and white digital images of fluorescent signals were processed using Fiji Image J, cropped and resized using Adobe Photoshop. Composite panels were generated using Adobe Illustrator.

Statistical analysis

Data were analysed for statistical significance (ANOVA, X^2 test and Fisher's exact test) using Prism6 (GraphPad Software) and online tools (www.quantpsy.org/chisq.htm, www.vassarstats.net/) and graphically illustrated using Excel (Microsoft) and Graph Pad Prism 6 (Graph Pad Software, Inc). Despite showing the same subregional distribution, the BBHX cell line showed line-to-line

variation with respect to dispersion metrics for graft progeny cell number and spread when compared to all other lines (hiPSC and hESC), denoting it as an outlier with respect to these outcomes. As such it was omitted from summaries of dispersion metrics for the hiPSC dataset.

Ethical review and approval

Appropriate ethical approval and patient consent were obtained for collection of human skin biopsies and derivation of human induced pluripotent stem cells (NHS North West Ethics Committee reference 13/NW/0205 and Addenbrooke's Hospital ethics reference 08/H0311/201; R&D No. A091485). Mouse embryos were obtained and cultured in accordance with the Animals (Scientific Procedures) Act 1986.

Supplemental References

Aflatoonian, B., Ruban, L., Shamsuddin, S., Baker, D., Andrews, P., and Moore, H. (2010). Generation of Sheffield (Shef) human embryonic stem cell lines using a microdrop culture system. In Vitro Cell. Dev. Biol. Anim. *46*, 236–241.

Beddington, S.P. (1981). An autoradiographic analysis of the potency of embryonic ectoderm in the 8th day postimplantation mouse embryo. J. Embryol. Exp. Morphol. *64*, 87–104.

Cho, C.H.-H., Hannan, N.R.-F., Docherty, F.M., Docherty, H.M., Joåo Lima, M., Trotter, M.W.B., Docherty, K., and Vallier, L. (2012). Inhibition of activin/nodal signalling is necessary for pancreatic differentiation of human pluripotent stem cells. Diabetologia *55*, 3284–3295.

Downs, K.M., and Davies, T. (1993). Staging of gastrulating mouse embryos by morphological landmarks in the dissecting microscope. Dev. Biol. *1266*, 1255–1266.

Fischer, A.H., Jacobson, K.A., Rose, J., and Zeller, R. (2008). Cryosectioning tissues. CSH Protoc. *2008*, pdb.prot4991.

Hannan, N.R.F., Segeritz, C.-P., Touboul, T., and Vallier, L. (2013). Production of hepatocyte-like cells from human pluripotent stem cells. Nat. Protoc. *8*, 430–437.

Rashid, S.T., Corbineau, S., Hannan, N., Marciniak, S.J., Miranda, E., Alexander, G., Huang-Doran, I., Griffin, J., Ahrlund-Richter, L., Skepper, J., et al. (2010). Modeling inherited metabolic disorders of the liver using human induced pluripotent stem cells. J. Clin. Invest. *120*, 3127–3136.

Takashima, Y., Guo, G., Loos, R., Nichols, J., Ficz, G., Krueger, F., Oxley, D., Santos, F., Clarke, J., Mansfield, W., et al. (2014). Resetting transcription factor control circuitry toward ground-state pluripotency in human. Cell *158*, 1254–1269.

Theiler, K. (1989). The House Mouse. Development and Normal Stages from Fertilization to 4 Weeks of Age (Berlin: Springer-Verlag).
